# Portal flow diversion based on portography is superior than puncture site in the prediction of overt hepatic encephalopathy after TIPS creation

**DOI:** 10.1186/s12876-022-02447-y

**Published:** 2022-07-29

**Authors:** Chongtu Yang, Yang Chen, Chaoyang Wang, Jiacheng Liu, Songjiang Huang, Chen Zhou, Yingliang Wang, Shuguang Ju, Tongqiang Li, Yaowei Bai, Wei Yao, Bin Xiong

**Affiliations:** 1 Department of Radiology, Union Hospital, Tongji Medical College, Huazhong University of Science and Technology, Jiefang Avenue #1277, Wuhan, 430022 China; 2 Hubei Province Key Laboratory of Molecular Imaging, Wuhan, 430022 China

**Keywords:** Cirrhosis, Portal hypertension, Portosystemic shunt, Transjugular intrahepatic, Hepatic encephalopathy, Portography

## Abstract

**Background:**

Targeted puncture of an appropriate portal venous branch during transjugular intrahepatic portosystemic shunt (TIPS) procedure may reduce the risk of postprocedural overt hepatic encephalopathy (HE). This study aimed to describe blood distribution under portography and combined it with puncture site to determine portal flow diversion, and to evaluate its prognostic value in predicting post-TIPS overt HE.

**Methods:**

In this retrospective analysis of patients with cirrhosis undergoing TIPS, we included 252 patients to describe blood distribution under portography and 243 patients to assess the association between portal flow diversion and post-TIPS overt HE.

**Results:**

At the first stage, 51 (20.2%) patients were identified as type A (unilateral type with the right portal branch receives blood from splenic vein [SV]), 16 (6.4%) as type B (unilateral type with the right branch receives blood from superior mesenteric vein [SMV]) and 185 (73.4%) as type C (fully mixed type). At the second stage, 40 patients were divided into the SV group, 25 into the SMV group and 178 into the mixed group. Compared with the mixed group, the risk of post-TIPS overt HE was significantly higher in the SMV group (adjusted HR 3.70 [95% CI 2.01–6.80]; *p* < 0.001), whereas the SV group showed a non-significantly decreased risk (adjusted HR 0.57 [95% CI 0.22–1.48]; *p* = 0.25). Additionally, the SMV group showed a substantial increase in ammonia level at 3 days and 1 month after procedure.

**Conclusions:**

Our results support the clinical use of portal flow diversion for risk stratification and decision-making in the management of post-TIPS overt HE.

**Supplementary Information:**

The online version contains supplementary material available at 10.1186/s12876-022-02447-y.

## Introduction

Currently, transjugular intrahepatic portosystemic shunt (TIPS) is recommended as a standard procedure for treating portal hypertension-related complications in patients with cirrhosis [1, 2]. However, the high prevalence of postprocedural overt hepatic encephalopathy (HE) limits the effectiveness and broad use of this procedure, with a reported incidence ranging from 10 to 50% within 1 year [3, 4].

Diversion of undetoxified portal blood into the systemic circulation is one of the major mechanisms involved in the pathogenesis of post-TIPS overt HE, which degree is related to the diameter and position of the stent. The association between stent diameter and overt HE has been well established [5, 6], while the effect of stent position on the outcome has not been fully elucidated. An early randomized-controlled trial (RCT) and few observational studies suggested that puncture of the left branch of portal vein (PV) might decrease the risk of post-TIPS overt HE compared to the right branch [7–13], and the potential mechanism might be related to the unbalanced distribution of blood flow from splenic vein (SV) and superior mesenteric vein (SMV) in the intrahepatic portal system. However, this theory lacks of hemodynamic evidence and may not be applicable to all cirrhotic patients due to anatomic variation.

During TIPS procedure, portography is performed to evaluate the portal system after access from the hepatic vein to the PV, which clearly demonstrates distribution of blood flow from the SMV and SV to the PV and its tributaries, and from a hemodynamic point of view, this approach is more accurate than assuming all patients have the same pattern of intrahepatic blood distribution. Thus, the first aim was to describe the distribution of intrahepatic portal blood from SMV and SV under intraprocedural portography, and the second aim was to combine portal blood distribution with puncture site to determine the type of portal flow diversion and evaluate its prognostic value in the prediction of post-TIPS overt HE in cirrhotic patients undergoing TIPS placement.

## Materials and methods

### Study population

From January 2016 to March 2021, all consecutive patients with portal hypertension admitted to receive TIPS creation were retrospectively analyzed. Baseline data regarding demographic characteristics, laboratory results and radiological findings were collected for each patient during hospitalization. Details of treatment were retrieved from electronic medical records. Follow-up at outpatient was scheduled for all patients at 1, 3, 6 and 12 months and then annually after procedure, supplemented with telephone interviews every 3 months. Patients were followed until death, liver transplantation or the end of the study (September 2021), and data were censored at the end of follow-up. The study protocol conforms to the ethical guidelines of the 1975 Declaration of Helsinki and was approved by the institutional ethics committee.

Patients with confirmed diagnosis of cirrhosis (clinical, radiologic or histologic) whom successfully undergoing TIPS were considered eligible for the study. Exclusion criteria were previous TIPS, advanced hepatocellular carcinoma (beyond Milan criteria) or other extrahepatic malignancy, presence of psychoactive drug intake or neurologic disorder, cavernous transformation of portal vein (CTPV) or portal vein thrombosis (PVT) with complete occlusion of left or right portal branch, and lost to follow-up within 3 months. In addition, we excluded patients with splenectomy history, with portography conducted through main PV (i.e. neither through the SV nor SMV) and with puncture of main portal venous bifurcation.

### Blood distribution and portal flow diversion

Details of TIPS procedure has been described previously [14]. In brief, access from the hepatic vein to PV were routinely established by blind fluoroscopic puncture. Afterwards, a hydrophilic wire and catheter were advanced to the SV or SMV (mostly the former) to perform the first portography to visualize the portal system. After dilating the tract with an angioplasty balloon and deploying a PTFE-covered stent, another portography was performed to confirm the stent position. Portography was obtained during a breath hold at the end of inspiration.

**Fig. 1:**
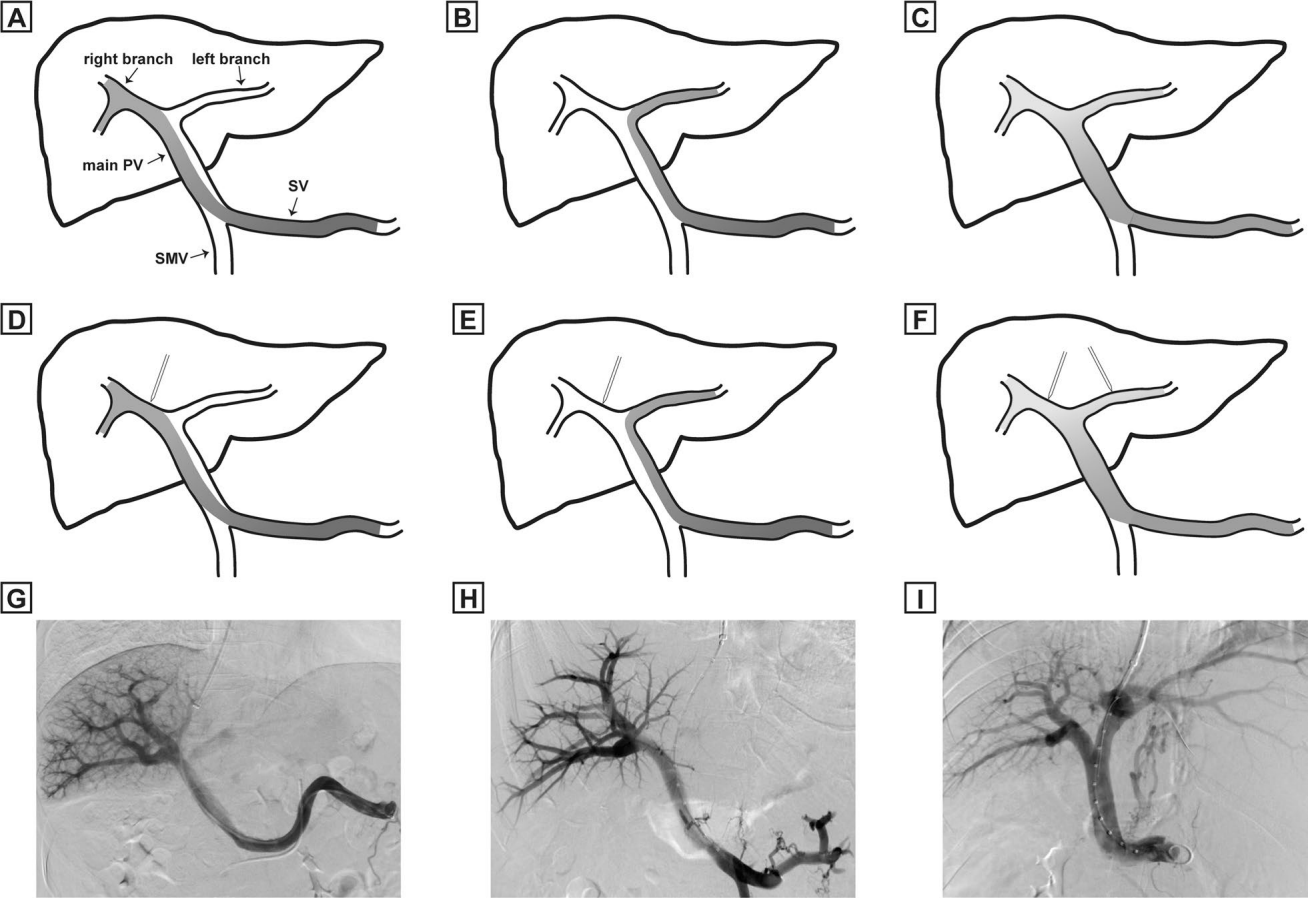
Blood distribution and portal flow diversion under portography. **A**, **B**, **C** Blood distribution. **A** Represents type A (unilateral type with the right portal branch receives blood from SV), **B** represents type B (unilateral type with the right branch receives blood from SMV), **C** represents type C (bilateral opacification). **D**, **E**, **F** Portal flow diversion. **D** Represents SV superiority type (TIPS shunt diverts blood from SV), **E** represents SMV superiority type (TIPS shunt diverts blood from SMV), **F** represents mixed type (type C regardless of the puncture site). **G**, **H**, **I** Flow diversion under portography during TIPS procedure. **G** Represents SV superiority type (case with type A and puncture of the right branch), **H** represents SMV superiority type (case with type A and puncture of the left branch), **I** represents mixed type (case with mixed type and puncture of the left branch). *SV* splenic vein, *SMV* superior mesenteric vein, *PV* portal vein

Blood distribution and flow diversion were determined by analyzing opacification of the portal venous branches using the first portography (before stent insertion). The first stage was to determine blood distribution from SV and SMV. Portography of the eligible patients were divided into two types: the unilateral type and bilateral type. The unilateral type indicates blood from SV and SMV were not fully mixed in the intrahepatic portal system (unilateral opacification of the PV under portography), and this type was further subdivided into the type A (the right branch receives blood from SV and the left branch receives blood from SMV) and type B (the right branch receives blood from SMV and the left branch receives blood from SV). The bilateral type (type C) indicates blood flow from SV and SMV were fully mixed (both left and right portal branch were opaque). The second stage was to determine portal flow diversion by taking puncture site into account. After excluding patients with puncturing the main portal venous bifurcation, the unilateral type were divided into the SV superiority type (SV group) and SMV superiority type (SMV group), with the former diverting blood from SV (puncture of the right branch for type A and the left branch for type B) and the latter diverting blood from SMV (puncture of the left branch for type A and the right branch for type B). Patients with bilateral type were classified as the mixed type (mixed group) regardless of puncturing the left or right branch (Fig. 1).

Blood distribution was determined by two researchers who were specialized in TIPS procedure. Both researchers independently assessed the DSA records of all patients, and resolved disagreements by discussion and making consensus. Inter-reviewer agreement was evaluated by kappa value calculation, which showed an excellent consistency in this study (kappa 0.879 [95% CI 0.811–0.945]).

### Endpoint definition

The primary endpoint of the study was incidence of post-TIPS overt HE. The spectrum of cognitive impairment occurred in a continuum and was subdivided into five grades according to the West Haven Criteria, only a grade II or higher was considered an episode of overt HE [4]. Due to the subjectivity of the diagnosis of overt HE, researchers responsible for the follow-up procedure were blinded to patients’ baseline characteristics. Secondary endpoints included changes of ammonia level at 3 days and 1 month after procedure.

### Statistical analysis

Quantitative variables are presented as means and SDs and categorical variables are presented as frequencies and percentages. Comparison between groups were conducted by Student t-test, Mann-Whiney test, Chi-squared test or Fisher’s exact test as appropriate.

Time-dependent endpoints were assessed by Kaplan–Meier curves and compared with log-rank tests. Hazard ratios (HRs) and 95% confidence intervals (95% CIs) for risk of the primary outcome were calculated with Cox proportional hazards models, and odds ratios (ORs) and 95% CIs for risk of secondary outcome were calculated with logistic regression models. Three models were built to adjust for potential confounders: in model A age and Child–Pugh score were adjusted; in model B age, Child–Pugh score and post-TIPS portal pressure gradient (PPG) were adjusted; in model C age, Child–Pugh score, post-TIPS PPG and puncture site (left or right branch) were adjusted. Additionally, two sets of subgroup analyses according to flow diversion and puncture site were conducted.

Sensitivity analysis based on Fine and Gray competing risk models were implemented, in which death and liver transplantation were considered as competing events for post-TIPS overt HE. For covariables included in the multivariable models, missing values were assumed to be missing at random, thereby were imputed using multiple imputations with chained equations. Statistical significance was set as *p* < 0.05 (two-sided). All analyses were performed using IBM SPSS (version 25.0) and R (version 4.0.3) with the add-on packages *mice, rms, survival* and *cmprsk*.

## Results

### Patient classification and general characteristics

**Fig. 2:**
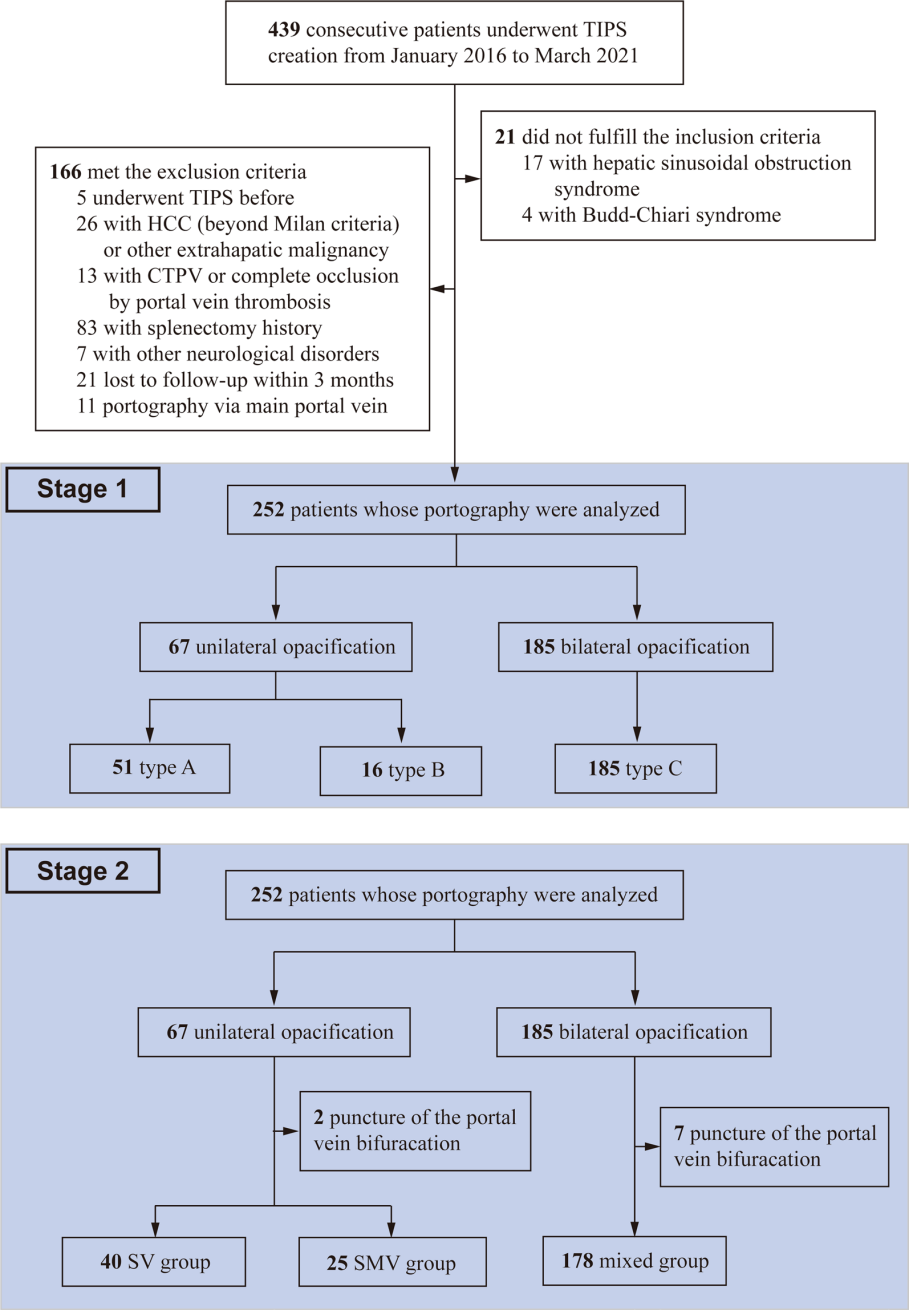
Flowchart of the patient selection protocol. *TIPS* transjugular intrahepatic portosystemic shunt, *HCC* hepatocellular carcinoma, *CTPV* cavernous transformation of portal vein, *SV* splenic vein, *SMV* superior mesenteric vein

Between January 2016 and March 2021, 439 consecutive patients scheduled to TIPS placement were screened retrospectively. At the first stage, 187 patients were excluded and for the remaining 252 patients, portography was analyzed to determine portal blood distribution, including 67 (26.6%) cases with unilateral opacification of the PV and the remaining 185 cases with bilateral opacification (type C). Among the 67 cases, 51 (76.1%) cases were further classified as type A and 16 cases were classified as type B (Fig. 2). Compared with type A and B, patients with the type C were more frequently female, with younger age, and treated TIPS for variceal bleeding.

At the second stage, 9 patients with puncture of portal venous bifurcation were further excluded and 243 patients were included to determine flow diversion. For 51 patients with type A, the right branch was punctured in 32 patients (SV group) and the left branch punctured in 19 patients (SMV group). For 14 patients with type B, the left branch was punctured in 8 patients (SV group) and the right branch punctured in 6 patients (SMV group) (Additional file 1: Fig. S1). Consequently, the final cohort included 40 (16.4%) patients in the SV group, 25 (10.3%) in the SMV group and 178 (73.3%) in the mixed group (Fig. 2). Baseline characteristics did not markedly differ among three groups, except for age (*p* = 0.002) and TIPS indication (*p* < 0.001). In addition, the SMV group tended to have higher Child–Pugh score (mainly derives from lower albumin level and more severe ascites), higher model for end-stage liver disease score (mainly derives from higher INR level) and lower pre- and post-TIPS PPG (Table 1).

**Table 1:** Baseline characteristics of the study population

	Total N = 243	SV group N = 40	SMV group N = 25	Mixed group N = 178	*P* value
*Demographics and clinical characteristics*					
Age*	54.6 (12.0)	54.2 (11.0)	62.5 (11.6)	53.6 (11.9)	0.002
Sex (Male)	155 (63.8)	26 (65.0)	20 (80.0)	109 (61.2)	0.185
Etiology					0.277
Virus related	168 (69.1)	28 (70.0)	14 (56.0)	126 (70.8)	
Alcohol related	22 (9.05)	5 (12.5)	2 (8.00)	15 (8.43)	
AIH	25 (10.3)	3 (7.50)	2 (8.00)	20 (11.2)	
Others	28 (11.5)	4 (10.0)	7 (28.0)	17 (9.55)	
TIPS indication					< 0.001
Variceal bleeding	212 (87.2)	31 (77.5)	17 (68.0)	164 (92.1)	
Refractory ascites	31 (12.8)	9 (22.5)	8 (32.0)	14 (7.87)	
Child–Pugh score*	7.53 (1.61)	7.15 (1.53)	7.96 (1.10)	7.55 (1.67)	0.131
Child–Pugh class					0.065
A	62 (26.2)	13 (32.5)	3 (12.0)	46 (26.7)	
B	153 (64.6)	25 (62.5)	22 (88.0)	106 (61.6)	
C	22 (9.28)	2 (5.00)	0 (0.00)	20 (11.6)	
MELD score*	11.9 (3.68)	11.4 (3.10)	13.0 (3.45)	11.9 (3.82)	0.238
MELD-Na score*	12.8 (4.71)	12.3 (4.40)	14.2 (4.77)	12.7 (4.76)	0.263
*Radiological findings*					
Ascites					0.005
Mild	84 (34.9)	10 (25.6)	3 (12.0)	71 (40.1)	
Moderate	39 (16.2)	9 (23.1)	5 (20.0)	25 (14.1)	
Severe	66 (27.4)	6 (15.4)	13 (52.0)	47 (26.6)	
Spontaneous porto-systemic shunt (Yes)	94 (38.7)	13 (32.5)	11 (44.0)	70 (39.3)	0.828
PV diameter (mm)*	15.4 (3.28)	14.5 (2.69)	15.3 (3.99)	15.6 (3.28)	0.172
Laboratory parameters*					
Bilirubin (µmol/L)	27.9 (28.3)	22.8 (11.4)	27.0 (13.1)	29.3 (32.3)	0.419
Albumin (g/L)	31.3 (5.53)	31.9 (5.37)	29.3 (5.53)	31.4 (5.54)	0.144
ALT (U/L)	37.1 (80.0)	32.8 (22.4)	37.6 (30.5)	38.0 (92.6)	0.935
Creatinine (µmol/L)	76.9 (60.6)	74.5 (27.3)	92.6 (75.7)	75.1 (63.7)	0.391
Sodium (mmol/L)	139 (4.43)	138 (3.94)	140 (5.61)	139 (4.36)	0.576
Prothrombin time (s)	16.8 (2.49)	16.5 (2.52)	17.0 (1.69)	16.8 (2.58)	0.703
INR	1.39 (0.26)	1.36 (0.26)	1.41 (0.18)	1.39 (0.27)	0.679
*TIPS procedure*					
Stent diameter					0.520
6 mm	108 (44.4)	19 (47.5)	12 (48.0)	77 (43.3)	
7 mm	87 (35.8)	16 (40.0)	6 (24.0)	65 (36.5)	
8 mm	48 (19.8)	5 (12.5)	7 (28.0)	36 (20.2)	
Pre-TIPS PPG*	36.8 (7.63)	34.8 (5.81)	33.7 (10.7)	37.5 (7.51)	0.080
Post-TIPS PPG*	15.9 (5.41)	15.3 (4.20)	13.1 (7.39)	16.3 (5.33)	0.088
Portography					0.169
Through SV	221 (90.9)	37 (92.5)	21 (84.0)	164 (92.1)	
Through SMV	22 (9.1)	3 (7.5)	5 (16.0)	14 (7.9)	
Puncture site					< 0.001
Left branch	112 (46.1)	7 (17.5)	17 (68.0)	88 (49.4)	
Right branch	131 (53.9)	33 (82.5)	8 (32.0)	90 (50.6)	
Embolization	166 (68.3)	29 (72.5)	15 (60.0)	122 (68.5)	0.569

*Data were expressed as means and standard deviation; Other data were presented as frequencies and percentages

*AIH* autoimmune hepatitis, *CTPV* cavernous transformation of portal vein, *MELD* model for end-stage liver disease, *PV* portal vein, *SV* splenic vein, *SMV* superior mesenteric vein, *ALT* alanine aminotransferase, *INR* international normalized ratio, *PPG* portal pressure gradient

### Primary endpoint

**Fig. 3:**
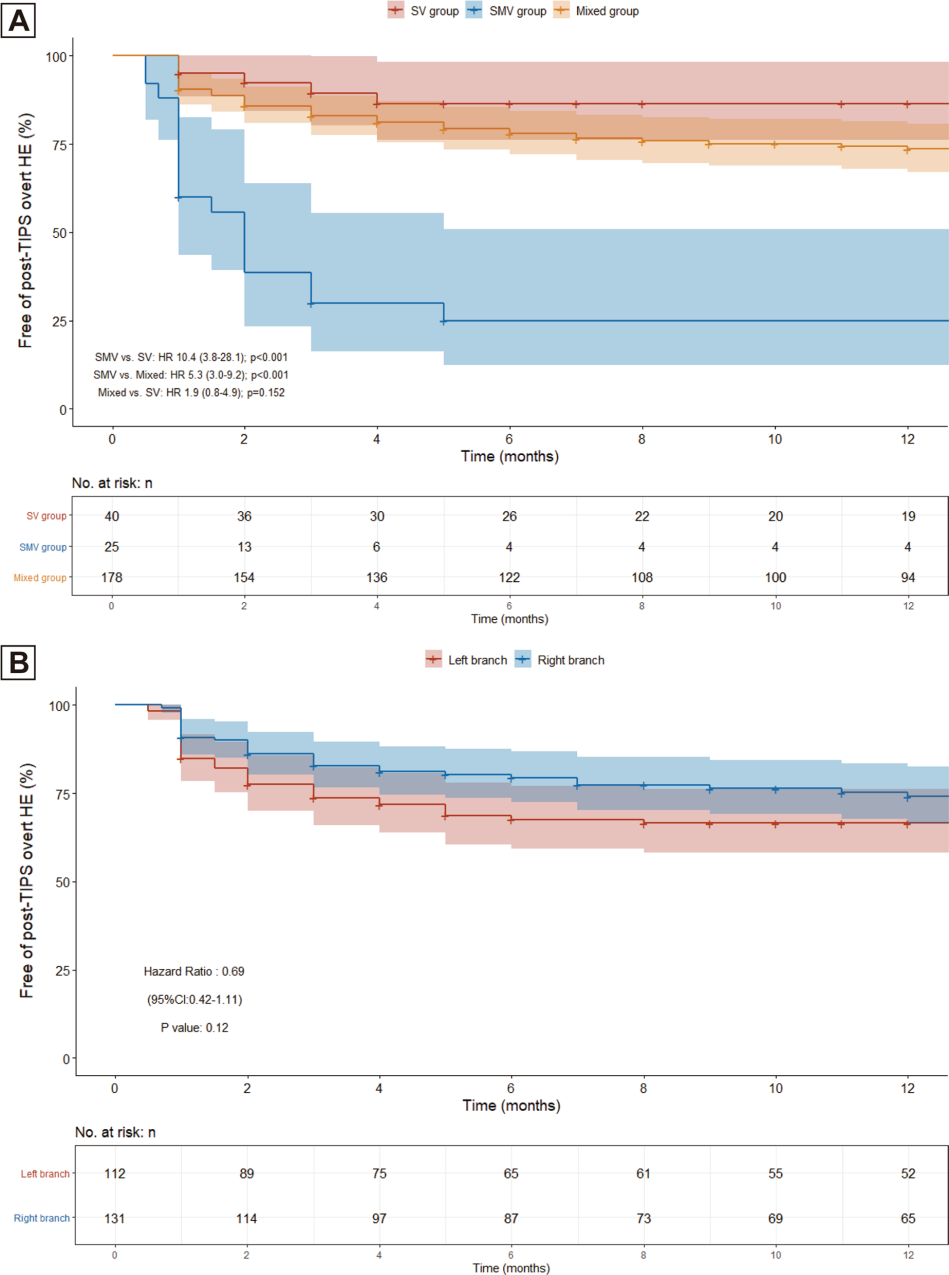
Post-TIPS overt hepatic encephalopathy in the study population. **A** Kaplan–Meier curves of patients stratified by flow diversion (SV group, SMV group and mixed group), **B** Kaplan–Meier curves of patients stratified by puncture site (left branch group and right branch group). *SV* splenic vein, *SMV* superior mesenteric vein, *HR* hazard ratio, *CI* confidence interval

During a median follow-up of 15.8 (IQR 9.4–26.5) months, 67 patients (27.6%) were identified to develop at least one episode of overt HE, including 8 patients (20.0%) in the SV group, 18 (72.0%) in the SMV group and 44 (24.7%) in the mixed group. Among them, 59 patients experienced a grade III or higher HE and 21 experienced more than one episode (Table 2). The cumulative rate of post-TIPS overt HE was significantly higher in the SMV group compared with the SV group (HR 10.4 [95% CI 3.8–28.1]; *p* < 0.001) and the mixed group (HR 5.3 [95% CI 3.0–9.2]; *p* < 0.001), but the risk did not markedly differ between the SV group and the mixed group (HR with mixed group: 1.9 [95% CI 0.8–4.9]; *p* = 0.152) (Fig. 3A). After adjusting for potential confounders, the presence of SMV superiority type was associated with a 2.7-fold increased risk of the outcome compared with the mixed type (adjusted HR 3.7 [95% CI 2.01–6.80]; *p* < 0.001), while the relative risk of the SV superiority type was 43% lower than the mixed type, though the difference was not statistically significant (adjusted HR 0.57 [95% CI 0.22–1.48]; *p* = 0.249) (model C in Table 3). In addition, sensitivity analysis based on Fine and Gray competing risk model showed consistent results (Additional file 1: Fig. S2).

**Table 2:** Summary of outcome measurements during follow-up

	Total N = 243	SV group N = 40	SMV group N = 25	Mixed group N = 178
Follow-up period (m)*	15.8 (9.4–26.5)	13.5 (5.7–26.2)	12.9 (4.7–21.4)	16.9 (5.7–28.3)
*Primary outcome: Incidence of post-TIPS overt HE*				
Overt HE	67 (27.6)	5 (12.5)	18 (72.0)	44 (24.7)
Grade III or higher	59 (24.2)	3 (7.5)	16 (64.0)	40 (22.4)
Recurrent	21 (8.6)	2 (5.0)	8 (32.0)	11 (6.2)
Precipitant				
High-protein intake	25 (37.3)	2 (40.0)	5 (27.8)	18 (40.9)
Rebleeding	9 (13.4)	0 (0.0)	2 (11.1)	7 (15.9)
Liver failure	18 (26.9)	2 (40.0)	7 (38.9)	9 (20.5)
Unclear	15 (22.4)	1 (20.0)	4 (22.2)	10 (22.7)
*Secondary outcome 1: Change of ammonia level at 3 days*^*†*^				
With ammonia elevation	81 (66.3)	12 (54.5)	17 (80.9)	52 (65.8)
Elevation level* (μmol/L)	9 (− 7–30)	4 (− 10–9)	31 (10–36)	8 (− 7–26)
*Secondary outcome 2: Change of ammonia level at 1 month*^*§*^				
With ammonia elevation	57 (76.0)	9 (75.0)	12 (92.3)	36 (72.0)
Elevation level* (μmol/L)	20 (4–52)	18 (1–30)	45 (23–60)	18 (− 1–55)

*Data were expressed as median (interquartile range); Other data were expressed as frequencies (percentages)

^†^For the secondary outcome 1, the results were available in 122 patients (22 patients for the SV group, 21 for the SMV group and 79 for the Mixed group)

^§^For the secondary outcome 2, the results were available in 75 patients (12 patients for the SV group, 13 for the SMV group and 50 for the Mixed group)

**Table 3:** Association between diversion type and incidence of outcomes

Diversion type	HR/OR (95% CI)		
	Model A	Model B	Model C
*Outcome 1: Development of overt HE*			
Mixed	Ref	Ref	Ref
SV superiority	0.57 (0.22–1.45)	0.58 (0.24–1.47)	0.57 (0.22–1.48)
SMV superiority	3.77 (2.09–6.78)	3.72 (2.05–6.75)	3.70 (2.01–6.80)
Outcome 2: Ammonia elevation at 3 days*			
Mixed	Ref	Ref	Ref
SV superiority	0.55 (0.20–1.51)	0.58 (0.21–1.59)	0.52 (0.18–1.48)
SMV superiority	2.94 (0.91–11.75)	2.98 (0.93–11.94)	3.21 (1.07–13.14)
Outcome 3: Ammonia elevation at 1 month*			
Mixed	Ref	Ref	Ref
SV superiority	1.05 (0.28–5.88)	1.10 (0.29–6.20)	1.02 (0.26–5.89)
SMV superiority	3.69 (0.57–73.81)	3.76 (0.58–75.43)	3.82 (0.59–76.94)

Model A was adjusted for age and Child–Pugh score

Model B was adjusted for age, Child–Pugh score and post-TIPS portal pressure gradient

Model C was adjusted for age, Child–Pugh score, post-TIPS portal pressure gradient and puncture site (left/right PV branch)

*For the secondary outcomes, effect of diversion type on the outcomes were evaluated by logistic regression models with odds ratios

*Ref* reference, *HR* hazard ratio, *OR* odds ratio, *CI* confidence interval, *HE* hepatic encephalopathy

### Secondary endpoints

**Fig. 4:**
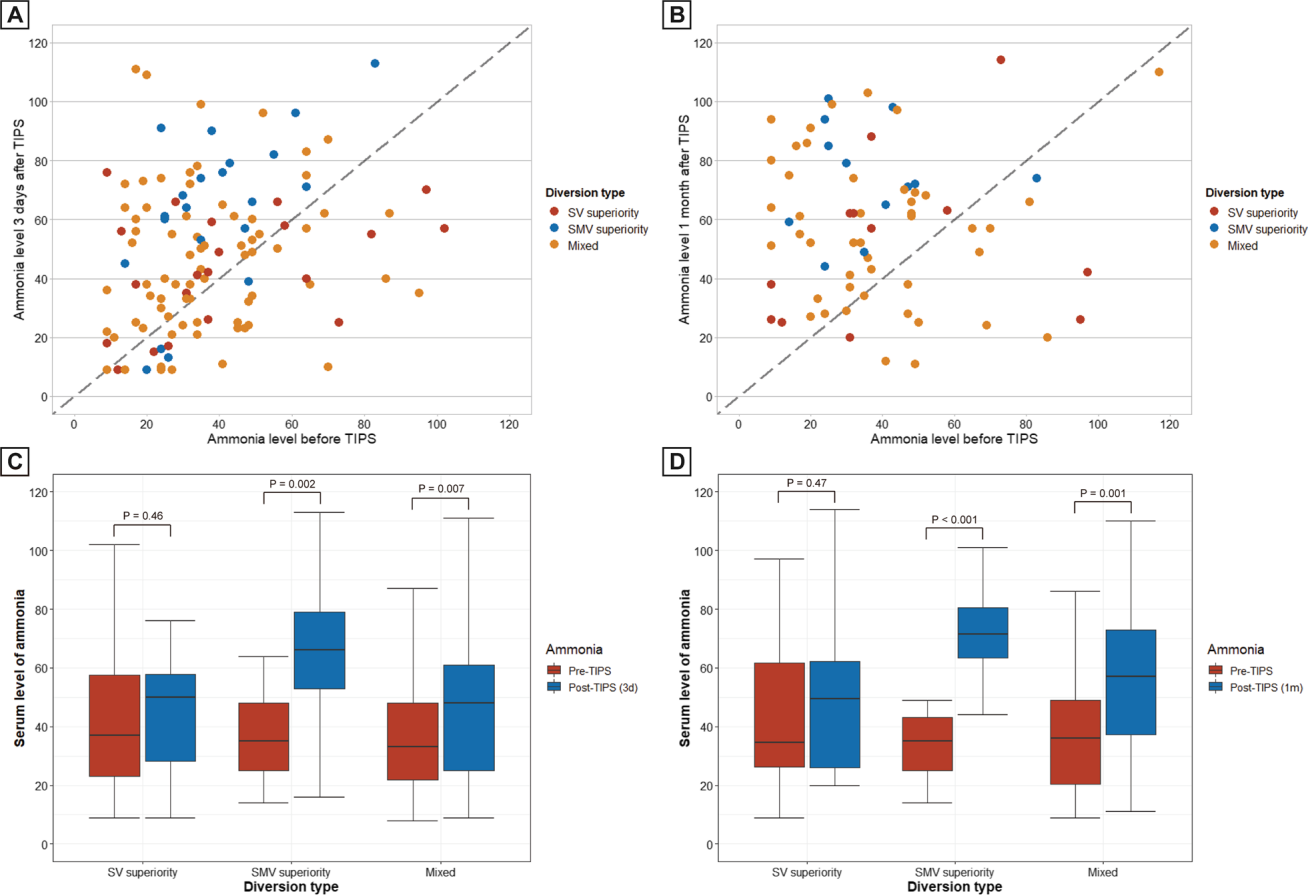
Change of ammonia level before and after TIPS creation. **A** and **C** Ammonia level before and at 3 days after procedure from 122 patients. **B** and **D** Ammonia level before and at 1 month after procedure from 75 patients. **A**, **B** In scatterplots, points below the diagonal line represent cases with the 3 days/1 month ammonia lever lower than the baseline level, and points above the diagonal line represent cases with the 3 days/1 month ammonia level higher than the baseline level. **C**, **D** Boxplots show the median (interquartile range) of ammonia level. P value were calculated using Mann–Whitney U tests

In the entire cohort, results of ammonia level before and at 3 days after TIPS creation were available in 122 (50.2%) patients, and results before and at 1 month were available in 75 (30.9%) patients. In the SV group, 12 (54.5%) and 9 (75.0%) patients experienced an increase in ammonia level at 3 days and 1 month, and the corresponding values were 17 (80.9%) and 12 (92.3%) in the SMV group, and 52 (65.8%) and 36 (72%) in the mixed group (Table 2, Fig. 4A, B). In the SMV group, median ammonia level increased significantly at 3 days (39 μmol/L [IQR 25–48] to 66 μmol/L [IQR 53–79]; *p* = 0.002) and 1 month (35 μmol/L [IQR 25–43] to 74 μmol/L [IQR 65–94]; *p* < 0.001). Similar pattern was observe in the mixed group (*p* = 0.007 and 0.001, respectively), but was not observed in the SV group (*p* = 0.46 and 0.47, respectively) (Fig. 4C, D). Compared with mixed type, the SMV superiority type was associated with a 2.2-fold increased risk of ammonia elevation at 3 days (adjusted HR 3.21 [95% CI 1.07–13.14]; *p* = 0.042), and a 2.8-fold increased risk at 1 month (adjusted HR 3.82 [95% CI 0.59–76.94]; *p* = 0.238), but the SV superiority type was only associated with a trend of decreased risk at 3 days (adjusted HR 0.52 [95% CI 0.18–1.48]; *p* = 0.223) (Table 3).

### Comparison between flow diversion and puncture site

We further evaluated the effect of puncture site on post-TIPS overt HE and compared its predictive ability with flow diversion. In our cohort, the cumulative rate showed no marked difference between puncture of the left and right portal branch (HR 0.69 [95% CI 0.42–1.11]; *p* = 0.12) (Fig. 3B). Similar results were obtained in the multivariable Cox analysis in which puncture site was not significantly correlated with the outcome (data not shown). In the subgroup analyses stratified by puncture site, the increased risk for the SMV superiority type was observed in both subgroups (Additional file 1: Fig. S3A), while in the subgroup analyses stratified by flow diversion, the cumulative rate of post-TIPS overt HE showed no marked difference between puncture of the left and right branch in all three subgroups (Additional file 1: Fig. S3B).

## Discussion

In this single-center, retrospective study with relatively large cohort of patients with cirrhosis submitted to TIPS, we found in approximately three quarters of cases the blood from SV and SMV were fully mixed in the intrahepatic portal system under portography, and these cases have comparable risk of postoperative overt HE regardless of the puncture site. For the remaining cases, approximately three quarters of them belong to type A (the right portal branch receives blood from SV and the left branch receives blood from SMV). Besides, portal flow diversion based on intraprocedural portography is an independent predictor of post-TIPS overt HE, in which the SMV superiority type is associated with increased risk of overt HE and ammonia elevation compared with the mixed type.

Hyperammonemia plays a central role in the pathogenesis of overt HE, especially after TIPS creation as this procedure diverts portal blood containing gut-derived neurotoxins into the systemic circulation [15, 16]. Thus, several studies concluded that puncture of the left portal branch might decrease the risk of post-TIPS overt HE based on the hypothesis that the left branch mainly receives blood from SV containing less neurotoxins [7, 11, 12]. However, this hypothesis was based on several animal studies [17, 18], and hemodynamic evidence from human studies (especially from patients with cirrhosis) were scarce. Contrary to previous views, our results showed that in 73.4% of patients with cirrhosis undergoing TIPS the blood from SV and SMV were fully mixed in the portal system, while the percentage of patients with the assumed blood distribution (type B) was only 5.6%. This discrepancy may be attribute to the fact that blood distribution depends on multiple factors including venous angles, blood velocity and length of the main PV. Thus, the previous hypothesis might not be applicable to all cirrhotic patients due to individual variation.

Based on these preliminary findings we subsequently combined blood distribution with puncture site to establish a new risk stratification approach based on portal flow diversion, and speculated that this approach is superior than using puncture site in the identification of patients at different risk of post-TIPS overt HE. In our cohort, patients presented with the SMV superiority type was associated with a significantly higher risk of the outcome, and the SV superiority type was related to a 43% decreased risk compared with the mixed type. Moreover, our hypothesis is supported by analyzing the change of serum ammonia level, in which the SMV group showed a substantially higher level within one month compared with the baseline level and the level of other two groups. Of note, we attempt to investigate the predictive value of flow diversion based on the portography after stent insertion, which might be more relevant to the outcome. However, portal flow was mostly diverted through TIPS shunt soon after stent placement and intrahepatic blood distribution was purely visual in some cases. Besides, the diversion degree in post-TIPS portography was affected by multiple factors (e.g. stent size and angle), making the results unreliable and reproduceable.

The cumulative rate of post-TIPS overt HE presented no difference between puncture of the left and right portal branch in the entire cohort as well as in subgroups of different flow diversion. This negative result could be explained on several grounds. First, most cases belonged to the mixed type which might not be affected by different puncture site. Second, previous studies suggested that puncture of the left branch has minimal impact on hepatic perfusion and less impairment on liver function since the left branch is smaller with fewer perfusion. However, atrophy of the right lobe and hypertrophy of the caudate and left lobe frequently occur in the presence of advanced cirrhosis [19–21]. Therefore, the effect of puncture site on post-TIPS overt HE is heterogeneous and flow diversion based on portography is a more reliable and accurate approach in the prediction of the outcome.

Our study is of clinical relevance as it reveals another independent risk factor for post-TIPS overt HE. For patients present with SMV superiority type under portography, under-dilated strategy to achieve a higher post-TIPS PPG may be an optimal choice [22, 23], and this subgroup of patients may benefit from pharmacological prophylaxis due to the positive results of the latest RCT [24]. Moreover, the incidence of overt HE in the SMV group was far beyond the reported ranges [4], and these patients were those with type A but were mis-punctured the left portal branch or type B but were mis-punctured the right branch. Since several modalities were developed to visualize the portal system which provides not only anatomical but hemodynamic information [25–27], novel techniques may aid clinicians to visualize blood distribution before procedure and select appropriate portal branch.

Potential limitations exist in the present study. First, patients in the SV group and SMV group were relatively small, limiting the ability to draw firm conclusions. Second, other outcomes such as shunt dysfunction and mortality were not assessed in our study, which were reported to have a correlation with different puncture site [9, 10, 13], though the results were controversial. Third, the reliability of identification of portal flow diversion may be confounded by different catheter position and different contrast dose, and standardized portography protocol should be generated. Forth, potential bias is inherent due to the retrospective nature and single-center design, and prospective studies are warranted to validate these results.

In conclusion, this study showed that in three quarters of patients submitted to TIPS the blood from SV and SMV were fully mixed in the intrahepatic portal system, and for patients with SMV superiority type the risk of post-TIPS overt HE increased significantly. Portal flow diversion based on portography is more reliable and accurate than puncture site in the stratification of patients at different risk of the outcome, which has the potential to affect decision-making by achieving preprocedural visualization of blood distribution.

## Supplementary Information

**Additional file 1. Fig. S1**. Venn diagram showing the distribution of patients with unilateral opacification stratified by blood distribution and flow diversion. SV splenic vein; SMV superior mesenteric vein.** Fig. S2**. Cumulative incidence of post-TIPS overt hepatic encephalopathy for patients with different flow diversion based on Fine-Gray competing risk models. P values were calculated by Fine-Gray tests. CI confidence interval; sHR subdistribution hazard ratio.** Fig. S3**. Primary outcome in different subgroups stratified by puncture site and flow diversion. (A) The association between flow diversion and post-TIPS overt hepatic encephalopathy in subgroups stratified by puncture site. (B) The association between puncture site and post-TIPS overt HE in subgroups stratified by flow diversion.

## Data Availability

The dataset used and analyzed in the present study are available from the corresponding author on reasonable request.

## Abbreviations

HE: Hepatic encephalopathy
TIPS: Transjugular intrahepatic portosystemic shunt
PV: Portal vein
SV: Splenic vein
SMV: Superior mesenteric vein
PPG: Portal pressure gradient
HR: Hazard ratio
OR: Odds ratio
MELD: Model for end-stage liver disease
