# Supplementary material for: Portal flow diversion based on portography is superior than puncture site in the prediction of overt hepatic encephalopathy after TIPS creation
Source: BMC Gastroenterol. 2022 Jul 29;22:363. doi: 10.1186/s12876-022-02447-y (PMC9336111; doi:10.1186/s12876-022-02447-y)
Supplement: Supplementary file 1 — Additional file 1. Fig. S1. Venn diagram showing the distribution of patients with unilateral opacification stratified by blood distribution and flow diversion. SV splenic vein; SMV superior mesenteric vein. Fig. S2. Cumulative incidence of post-TIPS overt hepatic encephalopathy for patients with different flow diversion based on Fine-Gray competing risk models. P values were calculated by Fine-Gray tests. CI confidence interval; sHR subdistribution hazard ratio. Fig. S3. Primary outcome in different subgroups stratified by puncture site and flow diversion. (A) The association between flow diversion and post-TIPS overt hepatic encephalopathy in subgroups stratified by puncture site. (B) The association between puncture site and post-TIPS overt HE in subgroups stratified by flow diversion. [file 12876_2022_2447_MOESM1_ESM.docx]

**Supplemental materials**

**Supplement figure 1.** Venn diagram showing the distribution of patients with unilateral opacification stratified by blood distribution and flow diversion. SV splenic vein; SMV superior mesenteric vein.


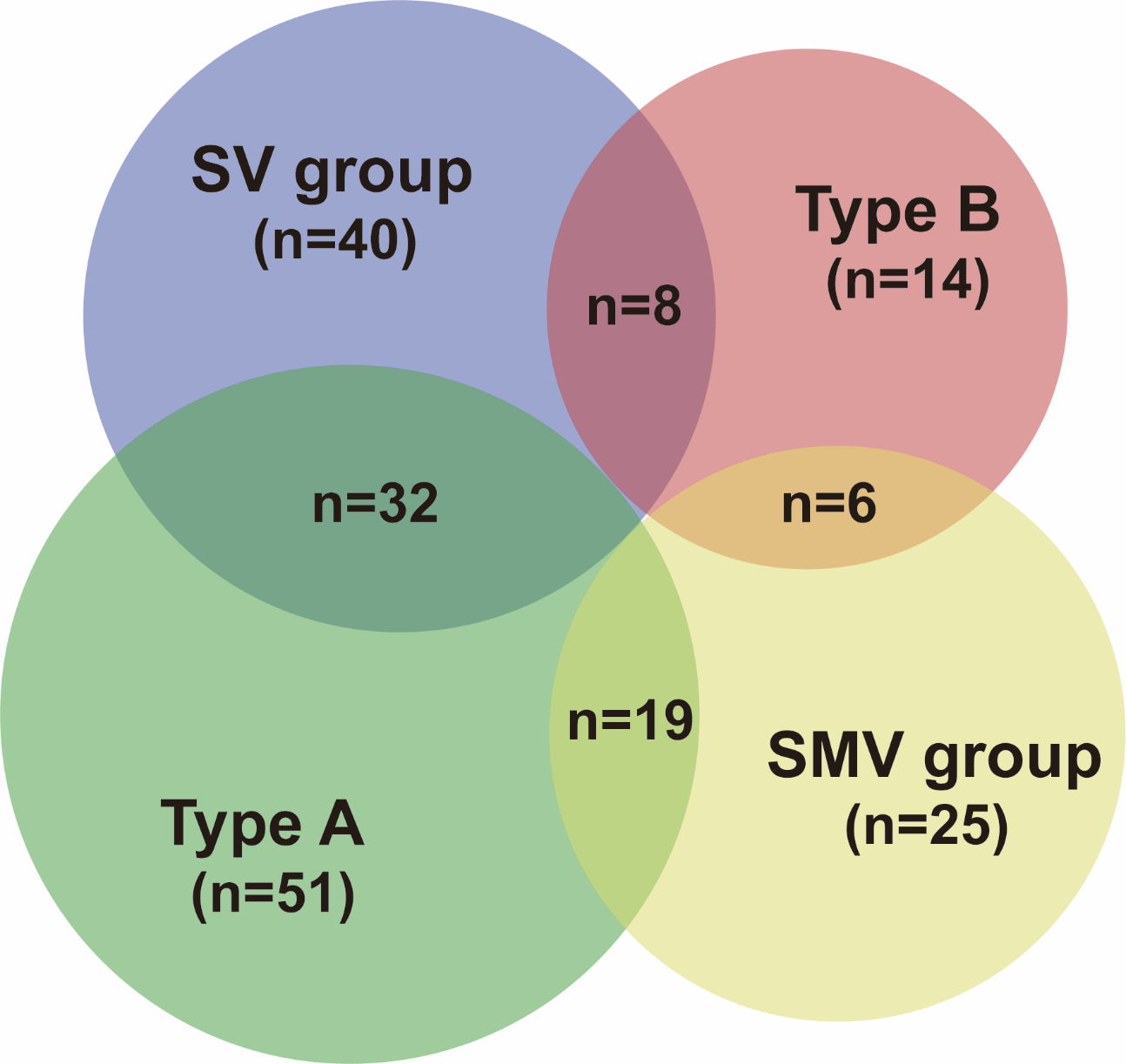


**Supplement figure 2.** Cumulative incidence of post-TIPS overt hepatic encephalopathy for patients with different flow diversion based on Fine-Gray competing risk models.

P value were calculated by Fine-Gray tests. CI confidence interval; sHR subdistribution hazard ratio.


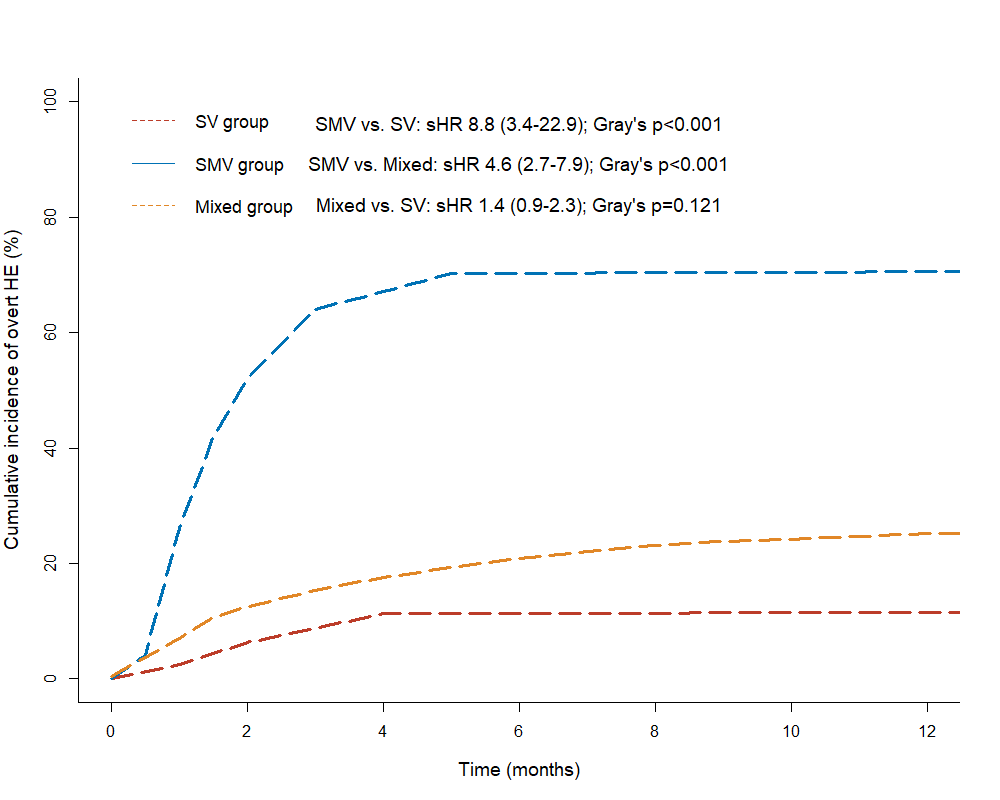


**Supplement figure 3.** Primary outcome in different subgroups stratified by puncture site and flow diversion.

**(A)** The association between flow diversion and post-TIPS overt hepatic encephalopathy in subgroups stratified by puncture site. **(B)** The association between puncture site and post-TIPS overt HE in subgroups stratified by flow diversion.

**
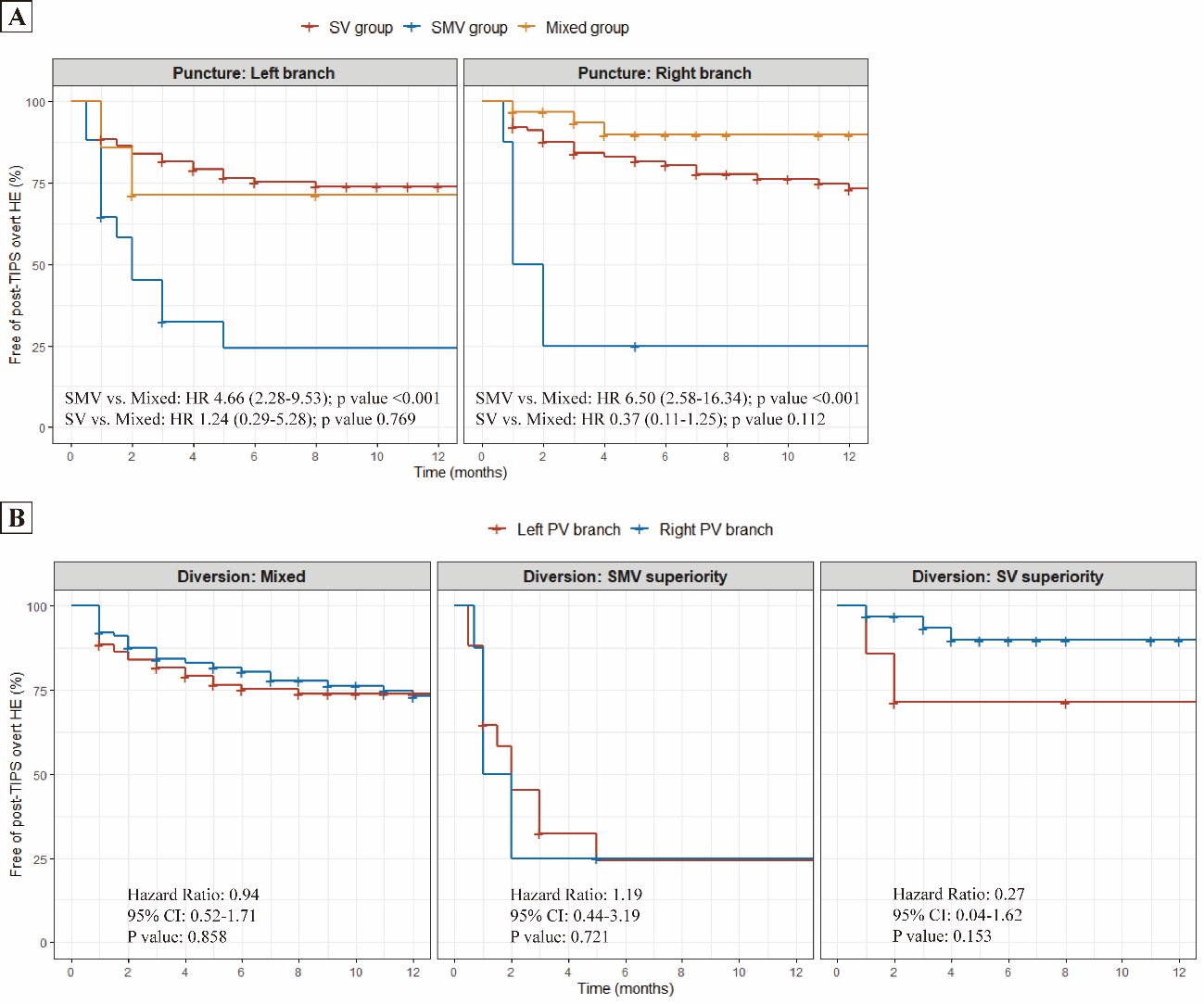
**
